# Zika virus infection in pregnancy and adverse fetal outcomes in São Paulo State, Brazil: a prospective cohort study

**DOI:** 10.1038/s41598-020-69235-0

**Published:** 2020-07-29

**Authors:** Nuria Sanchez Clemente, Elizabeth B. Brickley, Enny S. Paixão, Marcia F. De Almeida, Rosa E. Gazeta, Danila Vedovello, Laura C. Rodrigues, Steven S. Witkin, Saulo D. Passos

**Affiliations:** 1 Department of Epidemiology, University of São Paulo, São Paulo, Brazil; 2 Department of Infectious Disease Epidemiology, London School of Hygiene and Tropical Medicine, Keppel Street, London, WC1E 7HT UK; 3 Jundiaí Medical School, Jundiaí University, São Paulo, Brazil; 4 Department of Obstetrics and Gynecology, Weill Cornell Medicine, New York, NY USA; 5 Institute of Tropical Medicine, University of Sao Paulo Medical School, São Paulo, Brazil

**Keywords:** Epidemiology, Risk factors

## Abstract

Robust epidemiological and biological evidence supports a causal link between prenatal Zika Virus (ZIKV) infection and congenital brain abnormalities including microcephaly. However, it remains uncertain if ZIKV infection in pregnancy also increases the risk for other adverse fetal and birth outcomes. In a prospective cohort study we investigated the influence of ZIKV on the prevalence of prematurity, low birth weight, small-for-gestational-age, and fetal death as well as microcephaly (i.e., overall and disproportionate) in the offspring of women attending a high-risk pregnancy clinic during the recent ZIKV outbreak in Brazil. During the recruitment period (01 March 2016–23 August 2017), urine samples were tested for ZIKV by RT-PCR from all women attending the high-risk pregnancy clinic at Jundiaí University Hospital and from the neonates after delivery. Of the 574 women evaluated, 44 (7.7%) were ZIKV RT-PCR positive during pregnancy. Of the 409 neonates tested, 19 (4.6%) were ZIKV RT-PCR positive in the first 10 days of life. In this cohort, maternal ZIKV exposure was not associated with increased risks of prematurity, low birth weight, small-for-gestational-age, or fetal death. However, relative to ZIKV-negative neonates, ZIKV-positive infants had a five-fold increased risk of microcephaly overall (RR 5.1, 95% CI 1.2–22.5) and a ten-fold increased risk of disproportionate microcephaly (RR 10.3, 95% CI 2.0–52.6). Our findings provide new evidence that, in a high-risk pregnancy cohort, ZIKV RT-PCR positivity in the neonate at birth is strongly associated with microcephaly. However, ZIKV infection during pregnancy does not appear to influence the risks of prematurity, low birth weight, small-for-gestational-age or fetal death in women who already have gestational comorbidities. The results suggest disproportion between neonatal head circumference and weight may be a useful screening indicator for the detection of congenital microcephaly associated with ZIKV infection.

## Introduction

Zika Virus (ZIKV) has been detected sporadically in entomological and immunologic surveillance studies across Africa and Southeast Asia since the 1950s^1,2^. This mosquito-borne infection, which was previously considered to cause only mild disease^3^, spread to the Americas in 2014^4,5^. By August 2016, Brazil had reported 174,000 suspected and 78,500 confirmed cases of this flavivirosis, 5,500 of which were in the state of São Paulo^6–8^. The rapid dissemination of this single-stranded RNA virus and member of the Flaviviridae family^9^ in the Americas has been primarily attributed to the expansion of its principal vector, the mosquito *Aedes aegypti*^10^. ZIKV has also been shown to be transmitted sexually^11,12^ and has the potential to be transmitted in other body fluids, including breast milk^13,14^.

The association between maternal flavivirus infections and adverse birth outcomes has recently become more evident. For symptomatic dengue virus infections in pregnancy, a 2016 systematic review and meta-analysis^15^ reported positive associations with preterm delivery and low birth weight, while a matched case–control study in Brazil (2006–2012) found an increased risk of stillbirth^16^.

In the case of ZIKV, robust epidemiological and biological evidence now supports a causal link between prenatal ZIKV infection and a range of congenital brain abnormalities including microcephaly^17–21^. Case control study evidence from Recife in the Northeast of Brazil has shown that intra-uterine ZIKV exposure is associated with microcephaly^22,23^, and prospective cohort data in symptomatic women with suspected ZIKV in Rio de Janeiro have shown an association between congenital ZIKV infection and abnormal neurological examination and/or brain imaging at birth^24,25^, Studies quantifying the risk of other adverse fetal outcomes (i.e., prematurity, low birth weight, small-for-gestational age, and fetal death) associated with ZIKV infection during pregnancy are lacking.

Microcephaly occurs as the result of an insult that disturbs early brain growth^26^. In the case of ZIKV, microcephaly is associated with fetal brain disruption sequence (FBDS)^27,28^, a condition arising from a disturbance in brain tissue formation during the second or third trimester of pregnancy with subsequent fetal skull collapse resulting from decreased intracranial hydrostatic pressure^29^. Differentiation between proportionate and disproportionate microcephaly (i.e., disproportion between neonatal head circumference and weight) has been made in some ZIKV studies^25,30^, however its importance in helping to characterise the Congenital Zika Syndrome has still not been established.

The scientific community and the World Health Organization (WHO)^20^ have called for the urgent analysis of data from all ZIKV pregnancy cohorts to further understand the full spectrum of adverse outcomes associated with this infection in pregnancy; as well as maximise the availability of data for use in future meta-analyses^31^. In response to this call, this prospective cohort study in Jundiaí, São Paulo, compared the prevalence of five well-defined adverse fetal outcomes (i.e., prematurity, low birthweight, small-for-gestational-age, fetal death and microcephaly) in a group of ZIKV-positive and ZIKV-negative women and infants.

## Methods

### Study design and participants

This prospective cohort study from the Jundiaí Zika Cohort was initiated at Jundiaí University Hospital in São Paulo State, Brazil. The municipality of Jundiaí, located 60 km to the northwest of São Paulo city, has 409,000 inhabitants^32^ and one of the highest Human Development Indices of all the municipalities in the state^33^. The maternity department at the University Hospital is the only public maternity facility in the municipality and performs approximately 300–400 deliveries per month, an estimated two-thirds of the total births in Jundiaí^34^. The incidence and prevalence of ZIKV infection in Jundiaí during the study period has not been determined. However, in 2016, in the State of Sao Paulo, 9,845 cases of ZIKV were reported to the Brazilian Notifiable Disease Registry (SINAN)^35^. This would give a crude incidence of 0.2 cases of ZIKV per 1,000 inhabitants in the state of Sao Paulo in the year 2016. However, this is likely to be a gross underestimation as this figure only represents cases that accessed health services and were reported by health professionals, and, therefore, is likely to be biased to those with more severe clinical presentations.

During the recruitment period (1 March 2016–23 August 2017), all women attending the high-risk pregnancy clinic (i.e., due to the presence of risk factors threatening the life or health of the pregnant woman or her fetus)^36,37^ at Jundiaí University Hospital at any stage of pregnancy were considered eligible and offered the opportunity to participate in the study. Specifically, a pregnant woman was defined as high risk if she had sociodemographic risk factors (e.g., low or high BMI, extremes of age, drug use); had a previous adverse reproductive history (e.g., previous stillbirth, recurrent miscarriages); had a current obstetric illness (e.g., pre-eclampsia) or had an intercurrent disease during pregnancy (e.g., epilepsy, diabetes mellitus)^37^. The reasons for choosing this study population are explored in detail in our Cohort Profile^38^ but are summarised as follows (1) to try to maximise follow-up adherence, (2) to provide an appropriate location for the collection of samples and (3) to optimise newborn data by ensuring a large proportion would be born in Jundiai University Hospital. Over the duration of follow-up, clinical teams cared for pregnant women in accordance with standard Brazilian Ministry of Health protocols. At the time of enrolment, women of any stage of pregnancy were eligible to participate. The only exclusion criteria were women with life-threatening conditions and those with severe learning difficulties who could not give informed consent.

At enrolment, detailed demographic, medical and antenatal information, as well as examination findings were compiled by research nurses who interviewed the women and reviewed their antenatal records. Data was initially collected using an in-house data collection tool and subsequently, in August 2016, when a standardised tool was created by the WHO^39,40^, this was updated accordingly and immediately put into use. For the additional variables that were added from the WHO protocol, pregnant women who had been enrolled and had their baseline questionnaire administered prior to August 2016 were asked to answer any new questions, where possible, during follow-up visits. Data was entered digitally into the Cohort’s database (created using Salesforce™ Brasil online platform) and stored on a secured server.

Women had urine collected by research nurses at the time of recruitment and 2–3 weeks later for ZIKV detection. Subsequently, sample collection was repeated on a 2–3 monthly basis during routine antenatal visits. Trained volunteers carried out pre-arranged weekly follow-up telephone calls and asked the women whether they had experienced any symptoms consistent with ZIKV infection during that time. If symptoms were reported, the women were advised to report to the hospital for clinical review and collection of urine. Antenatal ultrasound scanning was carried out in months 3, 5, 7 and 8 in women who remained ZIKV negative and monthly in women who had a positive ZIKV RT-PCR and/or developed ZIKV-like symptoms during pregnancy according to the WHO definition^41^. All antenatal scans were carried out at the São Paulo Radiology Centre by sonographers specialising in fetal medicine and using Voluson 730 Expert/Voluson E6, GE equipment. At the time of delivery in hospital, both women and neonates had urine collected. Neonatal urine was collected using sterile urine bags.

### Laboratory procedures

All laboratory procedures were performed on de-identified samples. In the present study, due to severe financial constraints, ZIKV detection was limited to analysis by nucleic acid amplification testing of maternal and neonatal urine specimens collected during gestation and 10 days post-partum. Studies have shown that ZIKV is consistently detectable in urine for at least several weeks while its presence in serum is of shorter duration^9,42,43^.

For ZIKV detection, total RNA was extracted from urine by the commercial QIAamp Viral RNA Kit (Qiagen®), following the manufacturer’s instructions and stored at − 80 °C until used. ZIKV specific reverse transcription (RT) and quantitative polymerase chain reaction (qPCR) were performed with GoTaq® 1-Step RT-qPCR System (Promega®) on ABI Prism 7,500 SDS Real-Time cycler (Applied Biosystems). The ZIKV specific primers and probes designed by Lanciotti et al.^9^, are complementary to the non-structural 5 Protein (polymerase). The RT cycle consisted of a 10 min cycle at 50 °C and a 15 min cycle at 95 °C. The PCR consisted of forty cycles of 15 s at 95 °C and a 1 min cycle at 60 °C. Three positive controls (RNA extracted from positive ZIKV samples) and two negative controls (H_2_O) were included. We considered positive samples those that presented a threshold cycle (Ct) lower than 38.5 (as per Lanciotti et al.)^44^. In cases where the results were inconclusive, repetitions were performed with serially diluted samples.

### Anthropometric measures

Anthropometric measurements at birth (i.e., neonatal weight, length and head circumference) were obtained for all live-born infants, and the equipment used was consistent for all. Weight was assessed using digital scales, length using a recumbent baby length scale and head circumference using a standardised non-elastic tape measure. Z-scores for weight, length and head circumference were determined using the online Intergrowth calculator, which takes into account gestational age and sex^45–47^. Gestational age was estimated using first trimester ultrasound (USS) when available and by last menstrual period (LMP) when USS was unavailable. If USS or LMP-estimated gestational ages were not available, infant gender was missing, or anthropometric measures were not recorded at birth (i.e., if the neonate was delivered outside of the clinic), the infant and mother were excluded from the analytical cohort. Preterm birth was defined as any baby born alive before 37 completed weeks of pregnancy^48^. Low birth weight was defined as a birth weight of < 2500 g. Small-for-gestational-age (SGA) was defined as infants with birth weight z-scores of <  − 1.28 at birth (equivalent to 10th percentile) and extreme SGA as a birth weight of < − 1.88 z-scores (equivalent to the 3rd percentile)^49^. Microcephaly was defined as a head circumference z-score of < − 2 and severe microcephaly as a z-score < − 3^50,51^. Disproportionate microcephaly was defined, as per the National Birth Defects Network definition^51^, as a head circumference z-score of <  − 2 with a proportionally normal birth weight z-score of > − 2. Fetal loss was defined as a death prior to expulsion or complete extraction from the pregnant woman at any gestational age as per WHO/ICD-10^52^.

### Case definitions

Infants were considered to have been exposed to ZIKV during pregnancy if their mothers had at least one positive ZIKV RT-PCR urine sample during pregnancy and to have vertical ZIKV transmission if they had a positive ZIKV PCR urine sample within 10 days of birth^53,54^. Women were considered to be symptomatic for ZIKV if they met the WHO case definition for suspected ZIKV^41^, defined as a person presenting with rash and/or fever and at least one of the following signs or symptoms: arthralgia or arthritis or conjunctivitis (non-purulent/hyperaemic).

### Statistical analysis

The sample size was calculated using an estimated prevalence of cases of microcephaly among neonates of ZIKV RT-PCR positive pregnant women of 2%. A final analytical cohort size of n = 531 would give 80% power to detect a crude relative risk of two with a probability of type I error (α) of 5%. Although initially the Jundiai Zika Cohort aimed to enrol 500 pregnant women, recruitment continued for longer than initially anticipated to try to capture possible seasonal differences in the incidence of ZIKV disease. Criteria for the final selection of women to be included in the analytical cohort for this particular study were women with high-risk pregnancies who were tested for ZIKV using RT-PCR and whose babies had anthropometric measures at birth. Categorical variables were compared among women and infants with positive and negative ZIKV RT-PCR results using the Chi-square test except where there were less than 5 in any cell in which case Fisher’s exact test was used. Measures of association (Crude Risk Ratios) and 95% confidence intervals were calculated directly by comparing the prevalence of adverse fetal outcomes in the ZIKV exposed and unexposed groups. All statistical analyses were carried out using STATA™ version 15.1 software.

### Ethical approval and informed consent

This study received ethical approval by the research ethics committee of Jundiaí Medical School, protocol number 1446577 and methods were carried out specifically in accordance with the guidelines and regulations set thereby. Participating women provided written, informed consent for themselves and for future follow-up of their child.

## Results

### Study participants

**Figure 1:**
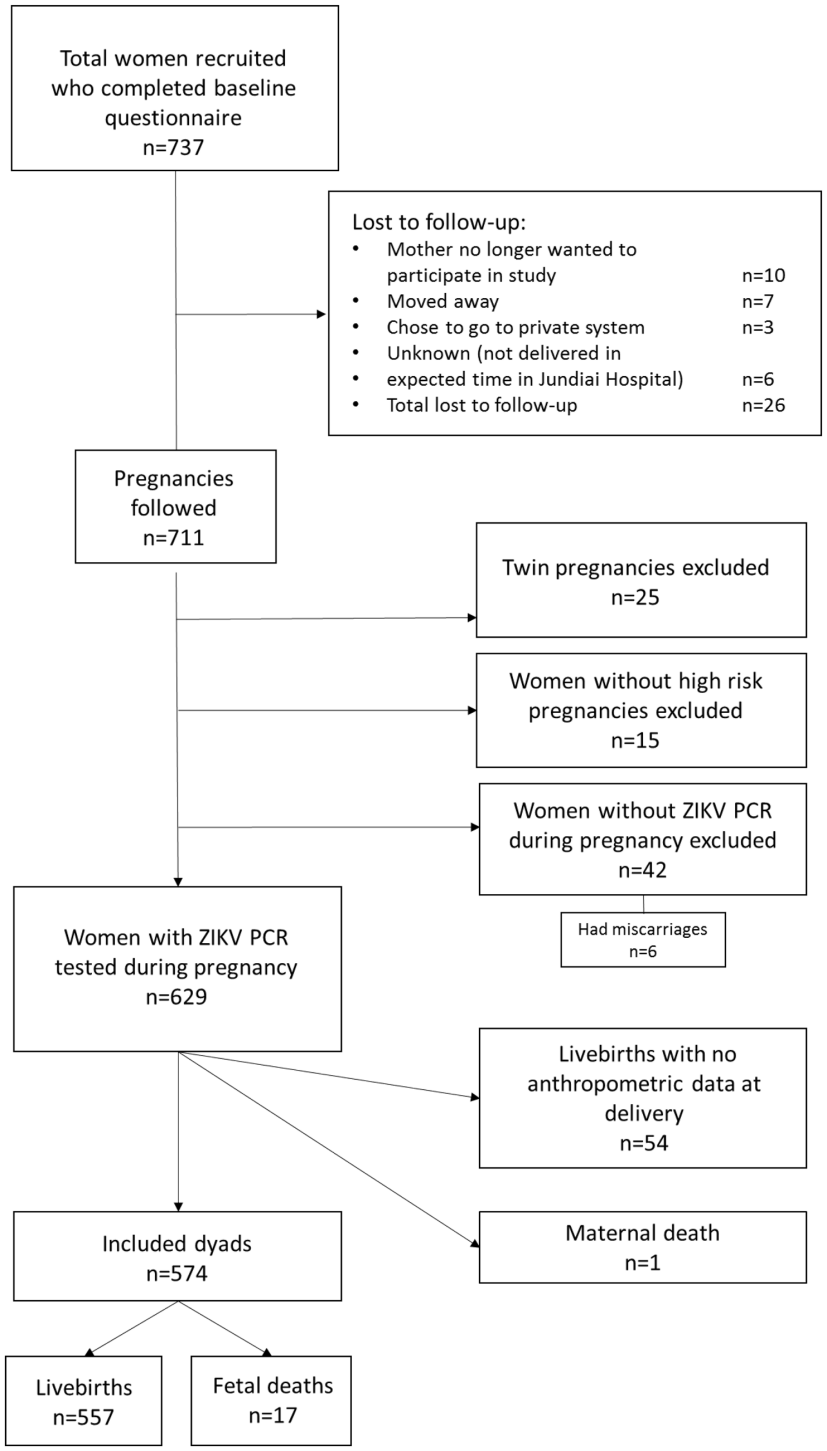
Flow diagram showing participants of the Jundiai Zika Cohort at each stage of the study, recruitment period: 01 March 2016–23 August 2017, Jundiai, São Paulo, Brazil.

A total of 737 women were initially enrolled in the study between March 2016 and August 2017 and completed the baseline questionnaire. Of these, 26 were lost to follow-up (see Fig. 1). Women with missing data pertaining to risk factors and outcomes of interest were also excluded from this analysis. In addition, for the purposes of answering this particular study question, twin pregnancies and 15 women who were recruited because of suspected ZIKV infection but who did not have other risk factors were excluded from the analysis. This resulted in 695 women who remained eligible and were followed. At the end of the follow-up period 62 women, including 6 who had miscarried, had not had a urine ZIKV RT-PCR test during pregnancy. Of the 618 women whose urine was ZIKV tested during pregnancy, 42 of their infants did not have anthropometric information collected at birth and there was one maternal death. This resulted in a total of 574 dyads, comprising 557 livebirths and 17 fetal deaths, in the analytical cohort (Fig. 1). ZIKV testing following delivery was available from 409 neonates, 71.3% of the maternal cohort.

During the 126.4 person-years of pregnancy follow-up time (mean 11.5 weeks, maximum 34.4 weeks) 44 women (7.7%) had a positive ZIKV urine test during pregnancy. The majority (61.4%, n = 27) tested positive in the 3rd trimester (36.4%, n = 16 in the 2nd trimester, 2.3%, n = 1 in the 1st trimester).

The baseline characteristics were similar between the women with and without detectable ZIKV infections in pregnancy (Table 1). In both groups, the majority of women were aged 20–34 years, had completed high school but had not undertaken any higher education studies. The ethnic make-up of the cohort is consistent with the demographic profile of the state of São Paulo^55^; approximately half of the women self-identified as white, a third mixed race (i.e., “parda”), 10% black, and around 2% indigenous or Asian. The majority women were married or lived with their partner; 60% reported that their pregnancy was unplanned. The cohort Caesarean section rate approached 50%, comparable to the Caesarean section rates in high-risk pregnancies in the public healthcare system in Brazil^56^. The majority of women were recruited in the 3rd trimester of pregnancy. Almost one third of the women had a diagnosis of diabetes and around 20% had hypertension during pregnancy.

**Table 1:** Maternal characteristics of participants of the Jundiaí Zika Cohort, March 2016 to August 2017, Jundiaí, São Paulo, Brazil.

	ZIKV RT-PCR positive women (n = 44)	ZIKV RT-PCR negative women (n = 530)	p-value^a^
**Age**			
13–19 years	10 (22.7%)	84 (15.9%)	0.47
20–34 years	26 (59.1%)	331 (62.5%)	
35–46 years	8 (18.2%)	115 (21.7%)	
Missing	0	0	
**Education**			
≤ 8 years	9 (20.9%)	90 (17.5%)	0.76
9–11 years	12 (27.3%)	118 (22.9%)	
12 years	16 (36.4%)	229 (44.5%)	
> 12 years	6 (14.0%)	78 (15.2%)	
Missing	1 (2.3%)	15 (2.8%)	
**Ethnicity/race**			
White	23 (52.3%)	278 (53.8%)	0.97*
Mixed race	16 (36.4%)	177 (34.2%)	
Black	4 (9.1%)	52 (10.1%)	
Other (Asian/indigenous)	1 (2.3%)	10 (1.9%)	
Missing	0	13 (2.5%)	
**Relationship with partner**			
Married/co-habiting	35 (79.6%)	395 (76.0%)	0.60
Single/divorced/widowed	9 (20.5%)	125 (24.0%)	
Missing	0	10 (1.9%)	
**Type of delivery**			
Vaginal/forceps	23 (52.3%)	257 (50.7%)	0.86
C-section	21 (47.7%)	250 (49.3%)	
Missing	0	23 (4.3%)	
**Trimester when recruited**			
1st	2 (4.7%)	26 (5.0%)	0.97*
2nd	15 (34.9%)	186 (35.8%)	
3rd	26 (60.5%)	308 (59.2%)	
Missing	1 (2.3%)	10 (1.9%)	
**Diabetes**			
Yes	14 (32.6%)	165 (32.7%)	0.99
No	29 (67.4%)	340 (67.3%)	
Missing	1 (2.3%)	25 (4.7%)	
**Hypertension**			
Yes	8 (18.2%)	102 (20.2%)	0.75
No	36 (81.8%)	403 (79.8%)	
Missing	0	25 (4.7%)	

Percentages for all categories were calculated with exclusion of those with missing data from the denominator.

^a^All p-values calculated using Chi^2^ test except for those labelled with asterisk which were calculated using Fisher’s exact test. The ‘missing’ category was not included as a category when the p-value was estimated.

### Fetal outcomes

In this high-risk pregnancy cohort, there was no significant difference in gestational age at birth between infants born to ZIKV positive or negative women (Table 2). Premature birth occurred in 9.1% of the ZIKV exposed and 13.3% of the unexposed women. Further, the proportion of early term (i.e., 37–38 weeks) and term and post-term (i.e., > 39 weeks) were similar in the exposed and unexposed groups.

**Table 2:** Gestational age at birth in weeks of live-born infants in the Jundiaí Zika Cohort March 2016 to August 2017, Jundiaí, São Paulo, Brazil.

Gestational age (completed weeks)	ZIKV exposed (n = 44)	ZIKV unexposed (n = 513)	Crude RR (95% CI)
< 37 (preterm)	4 (9.1%)	68 (13.3%)	0.7 (0.3–1.8)
37–38 (early term)	21 (47.7%)	222 (43.4%)	1.1 (0.8–1.5)
≥ 39 (term and post-term)	19 (43.2%)	222 (43.4%)	1.0 (0.7–1.4)
Missing	0	1 (0.2%)	

Low birth weight (< 2,500 g) was recorded for 9.1% of infants from ZIKV-positive women and 11.1% from ZIKV-negative women (Table 3). Defining SGA as birth weight z-score of <  − 1.28, 9.1% and 9.7% of infants from ZIKV-positve and negative women, met this definition respectively.

**Table 3:** Birth weight-related outcomes of infants born in the Jundiaí Zika Cohort from March 2016 to August 2017, Jundiaí, São Paulo, Brazil.

	Birth weight	ZIKV exposed (n = 44)	ZIKV unexposed (n = 513)	Crude RR (95% CI)
Birthweight	VLBW (< 1,500 g)	1 (2.3%)	10 (1.9%)	1.2 (0.2–8.9)
	LBW (1,500–2,499 g)	3 (6.8%)	47 (9.2%)	0.7 (0.24–2.3)
	Normal (2,500–4,000 g)	39 (88.6%)	435 (84.8%)	1.0 (0.9–1.2)
	Large (> 4,000 g)	1 (2.3%)	21 (4.1%)	0.6 (0.1–4.0)
	Total LBW	4 (9.1%)	57 (11.1%)	0.8 (0.3–2.1)
SGA	Extreme SGA (z-score < − 1.88)	2 (4.5%)	15 (2.9%)	1.6 (0.4–6.6)
	SGA (− 1.88 < z-score < − 1.28)	2 (4.5%)	36 (7.0%)	0.6 (0.2–2.6)
	Not SGA	40 (90.9%)	463 (90.3%)	1.0 (0.9–1.1)
	Total SGA	4 (9.1%)	50 (9.7%)	0.9 (0.4–2.5)

*VLBW* very low birth weight, *LBW* low birth weight, *SGA* small for gestational age (birth weight < − 1.28 z-scores).

All of the 17 women whose pregnancies ended in miscarriage or stillbirth, were ZIKV RT-PCR negative during gestation; it was not possible to obtain samples from these fetuses to determine their ZIKV status. Twelve infants (2.2%) were born with microcephaly, defined as a head circumference z-score of < − 2, and two (0.9%) of the infants had severe microcephaly (z-score < − 3). Microcephaly occurred in 4.5% infants born to ZIKV-positive women as compared to 1.9% of infants born to ZIKV-negative women (RR 2.3, 95% CI 0.5–10.3) (Table 4). Disproportionate microcephaly (defined as microcephaly with a birth weight z-score > − 2) occurred in two (4.5%) of the infants from ZIKV-positive women vs. versus five (1%) infants from ZIKV-negative women (RR 4.7 95% CI 0.9–23.3). Overall, adverse fetal outcomes occurred in 22.7% of pregnancies from ZIKV-positive women and 24.3% in ZIKV-negative women.

**Table 4:** Prevalence and relative risk of adverse outcomes among infants exposed (maternal ZIKV PCR positive) and unexposed to Zika Virus during pregnancy in the Jundiai Zika Cohort from March 2016 to August 2017, Jundiaí, SP, Brazil.

Variable	ZIKV RT-PCR positive women (n = 44)	ZIKV RT-PCR negative women (n = 513)	Crude RR (95% CI)
**All negative outcomes**	10 (22.7%)	129 (24.3%)	0.9 (0.5–1.6)
SGA	4 (9.1%)	50 (9.7%)	0.9 (0.4–2.5)
LBW	4 (9.1%)	57 (11.1%)	0.8 (0.3–2.1)
Microcephaly	2 (4.5%)	10 (1.9%)	2.3 (0.5–10.3)
Disproportionate	2 (4.5%)	5 (1.0%)	4.7 (0.9–23.3)
Proportionate	0	5 (0.8%)	–
Preterm	4 (9.1%)	68 (13.3%)	0.7 (0.3–1.8)
Fetal death	0	17 (3.3%)	–

Categories are not mutually exclusive. Microcephaly was defined as infants with head circumference z-scores < − 2 at birth. Severe microcephaly was defined as head circumference z-score of < − 3 at birth. Proportionate microcephaly was defined as infants with both head circumference and birth weight z-scores of < − 2 at birth and disproportionate microcephaly as head circumference z-score of < − 2 with birth weight z-score of > − 2. SGA = small for gestational age (birth weight < 10th percentile for sex and gestational age or < − 1.28 z-scores).

*LBW* low birth weight (birthweight < 2,500 g).

**Table 5:** Prevalence and relative risk of adverse outcomes among infants with presumed congenital Zika Virus infection (infant ZIKV PCR positive at birth) in the Jundiai Zika Cohort from March 2016 to August 2017, Jundiaí, SP, Brazil.

	Infant ZIKV RT-PCR positive at birth (n = 19)	Infant ZIKV RT-PCR negative at birth (n = 390)	Crude RR (95% CI)
**All adverse outcomes**	4 (21.1%)	86 (22.1%)	1.0 (0.4–2.3)
SGA	2 (10.5%)	42 (10.8%)	
LBW	2 (10.5%)	38 (9.7%)	(0.3–3.7)
Microcephaly	2 (10.5%)	8 (2.1%)	
Disproportionate	2 (10.5%)	4 (1.0%)	1.1 (0.3–4.1)
Proportionate	0	4	5.1 (1.2–22.5)
Preterm	1 (5.3%)	48 (12.3%)	10.3 (2.0–52.6)

Categories are not mutually exclusive. Microcephaly was defined as infants with head circumference z-scores of < − 2 at birth. Severe microcephaly was defined as head circumference z-score of < − 3 at birth. Proportionate microcephaly was defined as infants with both head circumference and birth weight z-scores of < − 2 at birth and disproportionate microcephaly as head circumference z-score of < − 2 with birth weight z-score of > − 2.

*SGA* small for gestational age (birth weight < 10th percentile for sex and gestational age or < − 1.28 z-scores), *LBW* low birth weight (birth weight < 2,500 g). Babies who had a positive ZIKV PCR within 10 days of birth were considered to be positive for this analysis.

For the 409 neonates with known ZIKV RT-PCR status, 19 (4.6%) had a positive ZIKV RT-PCR in the first 10 days of life. This analysis showed that the risk of microcephaly among neonates with detectable ZIKV RT-PCR at birth was five times the risk compared to the ZIKV-negative neonates (RR 5.1, 95% CI 1.2–22.5). Additionally, the risk of disproportionate microcephaly among neonates with positive ZIKV RT-PCR at birth was ten times the risk compared to neonates with negative ZIKV RT-PCR (RR 10.3, 95% CI 2.0–52.6). There were no statistical differences between the outcomes of preterm birth, low birth weight and SGA according to infant ZIKV RT-PCR status at birth. Overall, adverse outcomes occurred in 4 (21.1%) infants with positive ZIKV RT-PCR after birth compared to 86 (22.1%) infants with negative ZIKV RT-PCR after birth.

## Discussion

Using data collected prospectively in the Jundiaí Zika Cohort during the 2015–2017 outbreak of ZIKV in Brazil, the results of this study demonstrate that neonates who are positive for ZIKV in their urine within 10 days of birth have a fivefold greater risk of having microcephaly and a tenfold increased risk of having disproportionate microcephaly as compared to ZIKV-negative neonates. Conversely, we observed no differences in the risks of preterm birth, low birth weight or SGA in infants with or without detectable ZIKV in urine. These results aligned with our findings in comparing fetal and birth outcomes in the offspring of pregnant women with and without detectable ZIKV in their urine during pregnancy. Overall, our findings provide preliminary evidence that aside from the central nervous system sequelae, ZIKV does not appear to have substantive deleterious effects on pregnancy progression or fetal growth in high risk pregnancies.

Microcephaly was detected in a minority of ZIKV-positive neonates and in even a smaller percentage of babies from ZIKV-positive women. This low rate of progression to microcephaly is consistent with other studies carried out in Rio de Janeiro^25^, the French territories in the Americas^30^ and the USA^57–59^. Also similar to the Rio de Janeiro study, no significant differences were noted in our cohort between ZIKV-positive and negative groups in rates of preterm birth or SGA. In our study, fetal demise was a rare outcome that was only observed in ZIKV-negative women. Further analysis of large study populations and meta-analyses are needed to confirm that ZIKV is not typically a cause of fetal demise.

The strengths of our study are its prospective design, large sample size, utilization of a well-defined study population (attending a single high volume referral centre), the fact that women were recruited independently of them having ZIKV symptoms (providing us with a large comparison group) and the fact that all women received equivalent care under the same team of physicians.

The limitations of the study in part relate to the pressing nature of the ZIKV and microcephaly epidemic and the urgency to start the investigation, like many other cohorts in Brazil, prior to formal funding being secured.

The recruitment of only high-risk pregnant women brought advantages, as discussed earlier, in both logistics and maximisation of follow-up rates, however, these advantages also introduced limitations in external validity when generalising findings to the general pregnant population of Brazil. Moreover, as recruitment was carried out in a specific population of pregnant women that were users of a particular health service, it is possible that there are systematic differences between those recruited and those not recruited, which we were not able to measure, and these may have introduced bias. However, when comparing the sociodemographic profile of the pregnant women in our cohort and the profile of pregnant women living and using public maternity facilities in the State of São Paulo at the time of the study, we can see that they are quite similar. Additional comparative investigations are needed to determine if maternal and/or congenital ZIKV infection increases the risk of adverse pregnancy outcomes in standard risk pregnancies.

Another study limitation relates to the narrow window in which ZIKV can be detected in body fluids. In this study, urine samples were prioritised as they have been shown to be superior to blood samples in their window for ZIKV RT-PCR detection^9,42,43^. However, it is possible that some women in our cohort (particularly those who were asymptomatic) were indeed infected with ZIKV during their gestation but their viral load may have become undetectable by RT-PCR by the time their urine was collected and analysed. This may have contributed to the observed lack of concordance in ZIKV detection between women and their babies (Table S3) and the identification of microcephaly in babies from women who were ZIKV-negative during pregnancy. Overall, these results reinforce the conclusion that has also been reached by other studies, that the absence of ZIKV detection in urine and other biological fluids is not definitive for the absence of infection^60,61^. The concomitant use of serology, not available in the present investigation, may have provided additional validation of maternal exposure to this virus, although serological tests for ZIKV antibody also have significant inherent problems, notably cross-reactivity with other flaviviruses^62^, and the fact that individuals previously exposed to DENV do not mount a ZIKV IgM response^61^.

Ultimately, it is possible that this led to an underestimation of the risk of adverse outcomes related to ZIKV infection during pregnancy as the extent of exposure to ZIKV among pregnant women in the study may have been underestimated. However, if this was the case, our effects measures would have in fact been larger and more significant.

An additional limitation to our analysis is the absence of toxoplasmosis, rubella, cytomegalovirus, Herpes virus, syphilis, and parvovirus B19 (TORCH) antibody screening in our population. Negative findings would have offered stronger evidence that microcephaly in our cohort was due exclusively to ZIKV infection. However, a similar study carried out over the same time period in Paraíba in the Northeast of Brazil found that a substantial attributable risk of microcephaly was due to ZIKV (35–87% of microcephaly occurring during the time of the investigation was attributable to ZIKV)^63^.

In summary, our results show that microcephaly, and more specifically disproportionate microcephaly, is associated with congenital ZIKV infection. Nevertheless, exposure to ZIKV does not appear to increase the risk of other adverse fetal outcomes above the baseline risks observed in ZIKV-negative women with high risk pregnancies. The results suggest disproportion between neonatal head circumference and weight may be a useful screening indicator for detection of congenital microcephaly associated with ZIKV infection.

## Supplementary information

Supplementary Information.

## Data Availability

The data that support the findings of this study are available on request from the Principal Investigator [SP]. The data are not publicly available due to their containing information that could compromise the privacy of research participants.
