# Supplementary material for: Zika virus infection in pregnancy and adverse fetal outcomes in São Paulo State, Brazil: a prospective cohort study
Source: Sci Rep. 2020 Jul 29;10:12673. doi: 10.1038/s41598-020-69235-0 (PMC7391725; doi:10.1038/s41598-020-69235-0)
Supplement: Supplementary file 1 — Supplementary Information. [file 41598_2020_69235_MOESM1_ESM.pdf]

Zika Virus Infection in Pregnancy and Adverse Fetal Outcomes in São Paulo State, Brazil: A Prospective Cohort Study

Dr. Nuria SANCHEZ CLEMENTE*[1,2], Dr. Elizabeth B BRICKLEY[2], Dr Enny S PAIXÃO[2], Dr. Marcia F DE ALMEIDA[1], Dr. Rosa E GAZETA[3], Dr. Danila VEDOVELLO[3], Dr. Laura C RODRIGUES[2], Dr. Steven S. WITKIN[4], Jundiaí Zika Cohort Group¥, Dr. Saulo D PASSOS[3]

Supporting Information

S1 Checklist: STROBE Checklist

STROBE Statement—Checklist of items that should be included in reports of cohort studies

	Item No	Recommendation
Title and abstract	1	(a) Indicate the study's design with a commonly used term in the title or the abstract (Lines 1-3)
		(b) Provide in the abstract an informative and balanced summary of what was done and what was found (Lines 42-51)
Introduction		
Background/rationale	2	Explain the scientific background and rationale for the investigation being reported (Lines 89-101)
Objectives	3	State specific objectives, including any prespecified hypotheses (Lines 102-105)
Methods		
Study design	4	Present key elements of study design early in the paper (Line 108)
Setting	5	Describe the setting, locations, and relevant dates, including periods of recruitment, exposure, follow-up, and data collection (Lines 109-118)
Participants	6	(a) Give the eligibility criteria, and the sources and methods of selection of participants. Describe methods of follow-up (Lines 115-155)
		(b) For matched studies, give matching criteria and number of exposed and unexposed

Variables	7	Clearly define all outcomes, exposures, predictors, potential confounders, and effect modifiers. Give diagnostic criteria, if applicable (Lines 157-199)
Data sources/ measurement	8*	For each variable of interest, give sources of data and details of methods of assessment (measurement). Describe comparability of assessment methods if there is more than one group (Lines 157-167 (lab) and 181-186 (anthropometrics))
Bias	9	Describe any efforts to address potential sources of bias (Lines 126-129)
Study size	10	Explain how the study size was arrived at (Lines 201-203)
Quantitative variables	11	Explain how quantitative variables were handled in the analyses. If applicable, describe which groupings were chosen and why (Lines 169-180)
Statistical methods	12	(a) Describe all statistical methods, including those used to control for confounding (Lines 201-210)
		(b) Describe any methods used to examine subgroups and interactions (Lines 201-210)
		(c) Explain how missing data were addressed (See results tables)
		(d) If applicable, explain how loss to follow-up was addressed (Figure 2)
		(e) Describe any sensitivity analyses (N/A)
Results		
Participants	13*	(a) Report numbers of individuals at each stage of study—eg numbers potentially eligible, examined for eligibility, confirmed eligible, included in the study, completing follow-up, and analysed (Lines 217-225 and figure 2)
		(b) Give reasons for non-participation at each stage (Figure 2)
		(c) Consider use of a flow diagram (Figure 2)
Descriptive data	14*	(a) Give characteristics of study participants (eg demographic, clinical, social) and information on exposures and potential confounders (Table 1)
		(b) Indicate number of participants with missing data for each variable of interest (Tables 1-5)
		(c) Summarise follow-up time (eg, average and total amount) (Lines 228-229)
Outcome data	15*	Report numbers of outcome events or summary measures over time (Lines 229-232)

Main results	16	(a) Give unadjusted estimates and, if applicable, confounder-adjusted estimates and their precision (eg, 95% confidence interval). Make clear which confounders were adjusted for and why they were included (Tables 2-5)
		(b) Report category boundaries when continuous variables were categorized (Tables 1-5)
		(c) If relevant, consider translating estimates of relative risk into absolute risk for a meaningful time period
Other analyses	17	Report other analyses done—eg analyses of subgroups and interactions, and sensitivity analyses (Table 5)
Discussion		
Key results	18	Summarise key results with reference to study objectives (Lines 318-328)
Limitations	19	Discuss limitations of the study, taking into account sources of potential bias or imprecision. Discuss both direction and magnitude of any potential bias (Lines 353-396)
Interpretation	20	Give a cautious overall interpretation of results considering objectives, limitations, multiplicity of analyses, results from similar studies, and other relevant evidence (Lines 397-402)
Generalisability	21	Discuss the generalisability (external validity) of the study results (Lines 323, 397)
Other information		
Funding	22	Give the source of funding and the role of the funders for the present study and, if applicable, for the original study on which the present article is based (In the submission form)

*Give information separately for exposed and unexposed groups.

Note: An Explanation and Elaboration article discusses each checklist item and gives methodological background and published examples of transparent reporting. The STROBE checklist is best used in conjunction with this article (freely available on the Web sites of PLoS Medicine at http://www.plosmedicine.org/, Annals of Internal Medicine at http://www.annals.org/, and Epidemiology at http://www.epidem.com/). Information on the STROBE Initiative is available at http://www.strobe-statement.org

S2 Table 1. ZIKV RT-PCR results for mother-infant pairs in the Jundiai Zika Cohort

	Mother positive	Mother negative	Total
Baby positive	1	18	19
Baby negative	32	358	390
Baby untested	11	154	165
Total	44	530	574

S3 Table 2. Anthropometry of neonates born with microcephaly in the Jundiai Zika Cohort

Patient ID	Head circumference in cm (z-score)	Length in cm (z-score)	Weight in g (z-score)
80	31 (-2.47)	49.5 (0.07)	2600 (-1.67)
190	30 (-2.71)	44 (0.76)	2660 (-1.03)
197	30 (-2.46)	44 (-2.18)	2215 (-2.18)
199	29.5 (-2.77)	44 (-2.12)	3075 (0.44)
224	31.5 (-2.83)	47 (-2.17)	2595 (-2.35)
314	28.9 (-3.19)	41 (-3.3)	1690 (-2.87)
338	31.5 (-2.63)	47 (-3.07)	2750 (-1.07)
438	28 (-2.46)	40 (-2.52)	1420 (-2.19)
559	28 (-2.32)	42 (-1.4)	1995 (-0.37)
587	32 (-2.12)	47 (-1.87)	2790 (-1.67)
659	26.3 (-3.28)	37.5 (-2.92)	1300 (-1.89)
701	30.5 (-2.78)	45 (-2.38)	2125 (-2.66)
